# Induction of Protective Immunity against *Chlamydia muridarum* Intravaginal Infection with a Chlamydial Glycogen Phosphorylase

**DOI:** 10.1371/journal.pone.0032997

**Published:** 2012-03-12

**Authors:** Zhihong Li, Chunxue Lu, Bo Peng, Hao Zeng, Zhiguan Zhou, Yimou Wu, Guangming Zhong

**Affiliations:** 1 Department of Microbiology and Immunology, University of Texas Health Science Center at San Antonio, San Antonio, Texas, United States of America; 2 Department of Surgery, Second Xiangya Hospital, Central South University, Changsha, Hunan, China; 3 Department of Microbiology and Pathology, University of South China, Hengyang, Hunan, China; University of California Los Angeles, United States of America

## Abstract

We evaluated 7 *C. muridarum* ORFs for their ability to induce protection against chlamydial infection in a mouse intravaginal infection model. These antigens, although encoded in *C. muridarum* genome, are transcriptionally regulated by a cryptic plasmid that is known to contribute to *C. muridarum* pathogenesis. Of the 7 plasmid-regulated ORFs, the chlamydial glycogen phosphorylase or GlgP, when delivered into mice intramuscularly, induced the most pronounced protective immunity against *C. muridarum* intravaginal infection. The GlgP-immunized mice displayed a significant reduction in vaginal shedding of live organisms on day 14 after infection. The protection correlated well with a robust *C. muridarum*-specific antibody and a Th1-dominant T cell responses, which significantly reduced the severity but not overall incidence of hydrosalpinx. The GlgP-induced partial protection against upper genital tract pathology suggests that GlgP may be considered a component for a multi-subunit vaccine. These results have demonstrated that intramuscular immunization of mice with purified proteins can be used to identify vaccine antigens for preventing intravaginal infection with *C. trachomatis* in humans.

## Introduction

Urogenital tract infection with *Chlamydia trachomatis* is a leading cause of sexually transmitted bacterial infections [1], [2], [3]. Although antibiotics are effective in treating chlamydial infection, due to the lack of obvious symptoms, many infected individuals don't seek treatment, potentially leading to complications characterized with inflammatory pathologies in the upper genital tract, including pelvic inflammatory diseases, ectopic pregnancy and infertility [1], [4], [5]. Obviously, a most effective means to prevent *C. trachomatis*-induced complications is vaccination. However, there is still no licensed *C. trachomatis* vaccine [6]. Immunization with formalin-fixed whole *C. trachomatis* elementary body (EB) organisms not only failed to induce long lasting protective immunity against trachoma but also exacerbated ocular pathologies when some immunized children were exposed to natural infection [7]. These observations suggest that the formalin-fixed EB organisms may not only lack protective antigens or contain protective antigens in incorrect conformation but also carry pathogenic antigens. It is now known that many chlamydia-secreted proteins may not be retained in the EB organisms in any significant amounts [8], [9], [10], [11]. It has also been shown that the major outer membrane protein (MOMP) in its native conformation is more powerful than non-native MOMP in inducing protective immunity against chlamydial challenge infection [12], [13], [14]. More importantly, extensive immunological characterizations of chlamydial infection in animal models have demonstrated that chlamydial antigen-specific immune responses mediated by IFNg and dominated by Th1 T cells are essential for protection against chlamydial infection [6], [15], [16], Thus, it may be possible to induce protective immunity using the chlamydial antigens if prepared and delivered appropriately.

The precise pathogenic mechanisms of *C. trachomatis*-induced diseases remain unclear although both intracellular replication of *C. trachomatis* organisms and host responses to *C. trachomatis* antigens may significantly contribute to inflammatory pathologies during *C. trachomatis* infection [17], [18], [19], [20]. *C. muridarum* organisms deficient in a cryptic plasmid failed to induce pathologies in the upper genital tract while the wild type organisms were fully capable of doing so [20], [21]. Although *C. muridarum* organisms cause no known human diseases, the organisms have been extensively used to study immunobiology and search for vaccine antigens of *C. trachomatis* [6], [15], [16], [19], [20], [22], [23]. Extensive immunological studies, largely based on a *C. muridarum* intravaginal infection mouse model, have revealed that a Th1-dominant cell-mediated immunity is required for protection against Chlamydia urogenital tract infection [6], [15], [16]. The plasmid-free *C. trachomatis* organisms also displayed a reduced pathogenicity in mice [24]. The cryptic plasmid not only encodes 8 open reading frames (ORFs) of its own but also transcriptionally regulates 22 genomic ORFs [25]. It has been hypothesized that the plasmid-encoded and/or -regulated ORFs may contribute to chlamydial pathogenesis.

In the current study, we used a *C. muridarum* intravaginal infection mouse model to identify chlamydial antigens that can induce protective immunity against chlamydial intravaginal infection. We focused on chlamydial plasmid-regulated ORFs in the current study because plasmid-encoded and/or regulated genes have been proposed to play important roles in chlamydial pathogenesis [21], [25]. Among the 22 plasmid-regulated gene products, 7 *C. trachomatis* proteins were significantly recognized by *C. trachomatis*-infected women [26]. Since our long-term goal is to develop a vaccine for humans, we compared the protection efficacy of these 7 antigens using their orthologs from *C. muridarum* in the mouse model. We found that the chlamydial glycogen phosphorylase (GlgP), when delivered into mice intramuscularly, induced the most pronounced protective immunity that partially protected mice from chlamydial live organism infection and the infection-induced upper genital tract pathology. The partial protection further correlated with robust *C. muridarum*-specific antibody and Th1-dominant T cell responses.

## Results

### 1. Immunization with glycogen phosphorylase significantly reduced live organism shedding following an intravaginal infection

**Figure 1:**
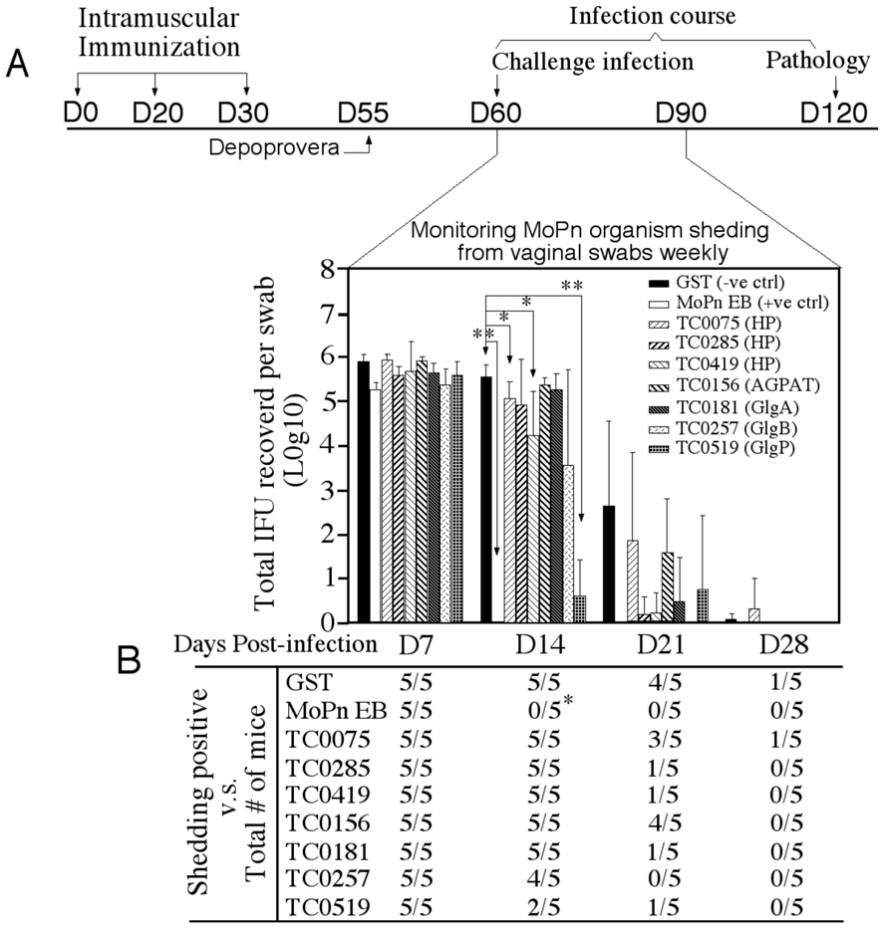
Evaluation of 7 plasmid-regulated antigens for inducing protective immunity against *C. muridaum* intravaginal infection. (A) Groups of female Balb/c mice with 5/each were immunized intramuscularly with three doses of corresponding antigens plus adjuvant (CpG+IFA) as indicated in the figure. One month following the final immunization, mice were challenged intravaginally with 2×10^4^ IFUs of *C. muridarum* organisms. Vaginal swabs were taken weekly as indicated along the x-axis for measuring the number of live organisms (IFUs). The IFUs from each swab was converted into Log10, and the log10 IFUs were used to calculate mean and standard deviation for each mouse group as displayed along the y-axis. The log10 IFUs were compared between 9 groups of mice at each time point using ANOVA test, followed by a two-tailed Student's *t*-test for comparing between the GST control group and a test antigen group. ** indicates p<0.01 while *, p<0.05. Although three antigen groups (TC0075, TC0419 & TC0519 or GlgP) displayed significant reduction in live organism shedding, only the GlgP-immunized group maintains a statistic difference after correcting multiple group comparison. (B) Number of mice with detectable infectious units (IFUs) from each group and at each time point was listed. The rates of IFU positivity were compared between the GST control and a test group using the Fisher's exact test. * indicates p<0.05. Three of the 5 mice immunized with GlgP cleared infection on day 14 post infection.

The chlamydial plasmid is known to transcriptionally regulate at least 22 chlamydial chromosomal genes [25]. We screened 7 of the plasmid-regulated gene products in a mouse intravaginal infection model for induction of protective immunity (Fig. 1). The 7 proteins include 3 hypothetical proteins with no known function (HP; ORFs TC0075, TC0285 & TC0419), 3 glycogen metabolism enzymes [glycogen synthase (GlgA, TC0181), glycogen branching enzyme (GlgB, TC0257) & glycogen phosphorylase (GlgP, TC0519)] and one acyltransferase (1-acyl-sn-glycerol-3-phosphate acyltransferase or AGPAT, TC0156). The initial screening experiment included 5 mice in each group. Despite the small group size, the positive control group immunized with *C. muridarum* live organisms (MoPn EBs) completely cleared infection on day 14 (p<0.01 when IFUs were compared, panel A; p<0.05 when the number of mice with positive shedding was compared, panel B). More importantly, the mouse groups immunized with the hypothetical proteins TC0075 or TC0419 or glycogen phosphorylase (GlgP) also displayed statistically significant reduction in shedding of live organisms on day 14 post infection. However, after correcting for multiple group comparisons, the p value only from the GlgP-immunized group remained statistically significant.

### 2. GlgP-immunized mice displayed significantly less severe hydrosalpinx

**Figure 2:**
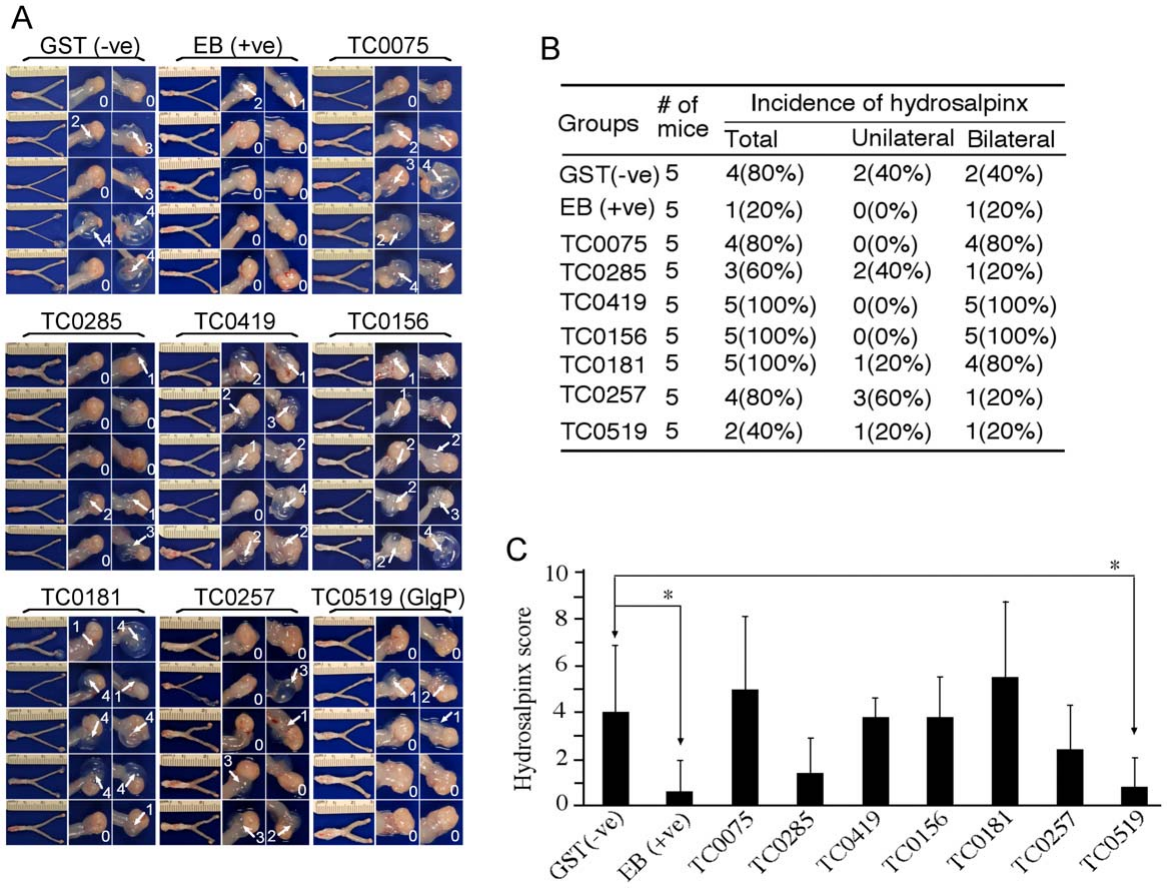
Effect of immunization on genital tract pathologies induced by intravaginal chlamydial infection. The 9 groups of mice described in Fig. 1 legend were sacrificed for evaluating pathologies of the mouse genital tract tissues. (**A**) The genital tract gross appearance images from all 9 groups of mice were presented with each group marked with the name of the corresponding antigens used to immunize the groups. The entire genital tract from vagina to ovary was displayed from left to right (left panels) and the oviduct and ovary regions were amplified (right panels). Each oviduct was scored for hydrosalpinx severity under naked eye and a numerical score was assigned for each oviduct as shown in the corresponding images. The scoring criteria were described in the method section. (**B**) The number of mice with unilateral or bilateral hydrosalpinx from each group was summarized. Note that 4 of 5 mice in GST while only 1 of 5 in EB-immunized groups developed hydrosalpinx. (**C**) The hydrosalpinx severity scores from each group of mice (indicated along the x-axis) were displayed along the Y-axis. Note that both the EB- and GlgP-immunized groups developed significantly reduced oviduct pathologies. The hydrosalpinx severity scores were compared between different groups using ANOVA followed by the two-tailed Student *t* test. * indicates p<0.05.

**Figure 3:**
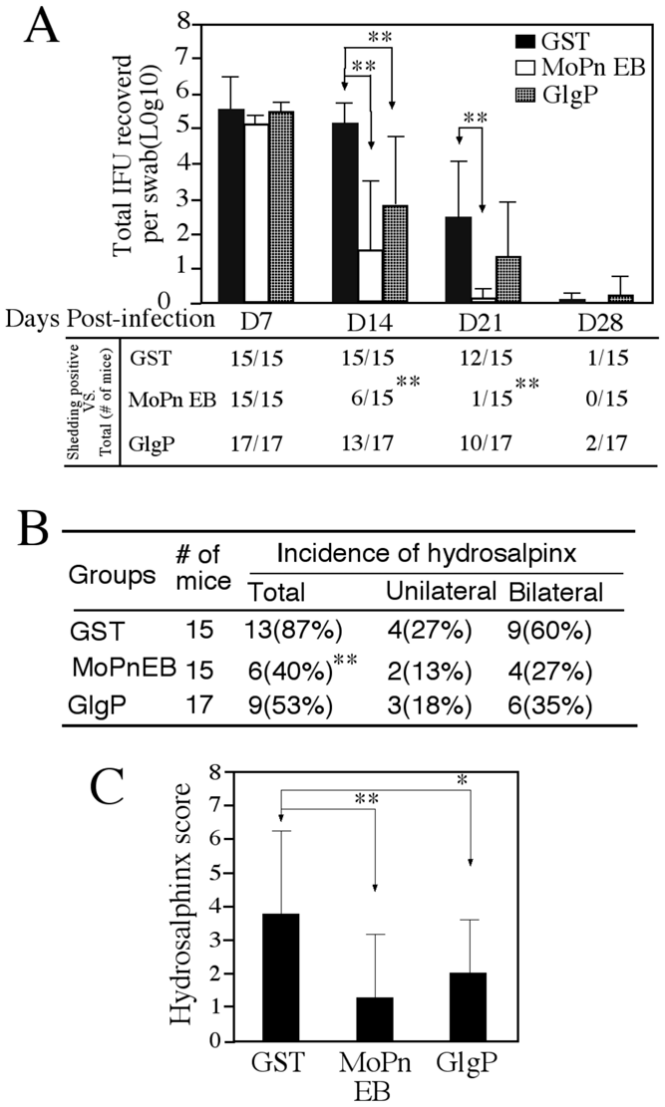
Enhanced resolution of *C. muridarum* genital tract infection by GlgP immunization. GlgP along with two control groups (GST as negative and EB as positive controls) of mice (15∼17mice/group) were immunized and infected as described in Fig. 1 legend. (**A**) Both the EB- and GlgP-immunized groups displayed significantly reduced levels of live organism shedding on day 14 postinfection and 9 of 15 mice from the EB-immunized group cleared infection on this day. (**B**) The incidence of hydrosalpinx from the 3 groups of mice was summarized. (**C**) The hydrosalpinx scores from each group were displayed along the Y-axis. ** indicates p<0.01 while * for p<0.05. The quantitative IFU and semi-quantitative hydrosalpinx score data were analyzed with Student *t* test while number of mice with positive IFUs or with hydrosalpinx were analyzed with Fisher's Exact Test.

We also monitored the pathologies in the upper genital tract tissues of the mice described above. The entire urogenital tracts were visually inspected for both the presence of hydrosalpinx and severity of hydrosalpinx based on the size of swollen oviducts relative to the size of ovary on the same side as described previously [19], [20], [27]. The overall incidences of hydrosalpinx were similar between the GlgP-immunized and GST control groups although the EB immunization group developed significantly lower rates of hydrosalpinx (Fig. 2 panels A & B). Importantly, when the severity of hydrosalpinx was compared, the GlgP-immunized group developed significantly less severe hydrosalpinx comparing to the GST control group (Fig. 2C). This reduced severity of hydrosalpinx in the GlgP-immunized group correlated well with reduced inflammatory infiltration examined under microscope (data not shown). The GlgP-induced protection was reproduced when more than 15 mice per group were used (Fig. 3). Thus, GlgP immunization not only decreased the live organism shedding from the lower genital tract but also reduced pathologies in the upper genital tract following a challenge infection with *C. muridarum* organisms.

### 3. Immunization with GlgP induced *C. muridarum*-specific immune responses with a minimal cross-reactivity with host GlgP

**Figure 4:**
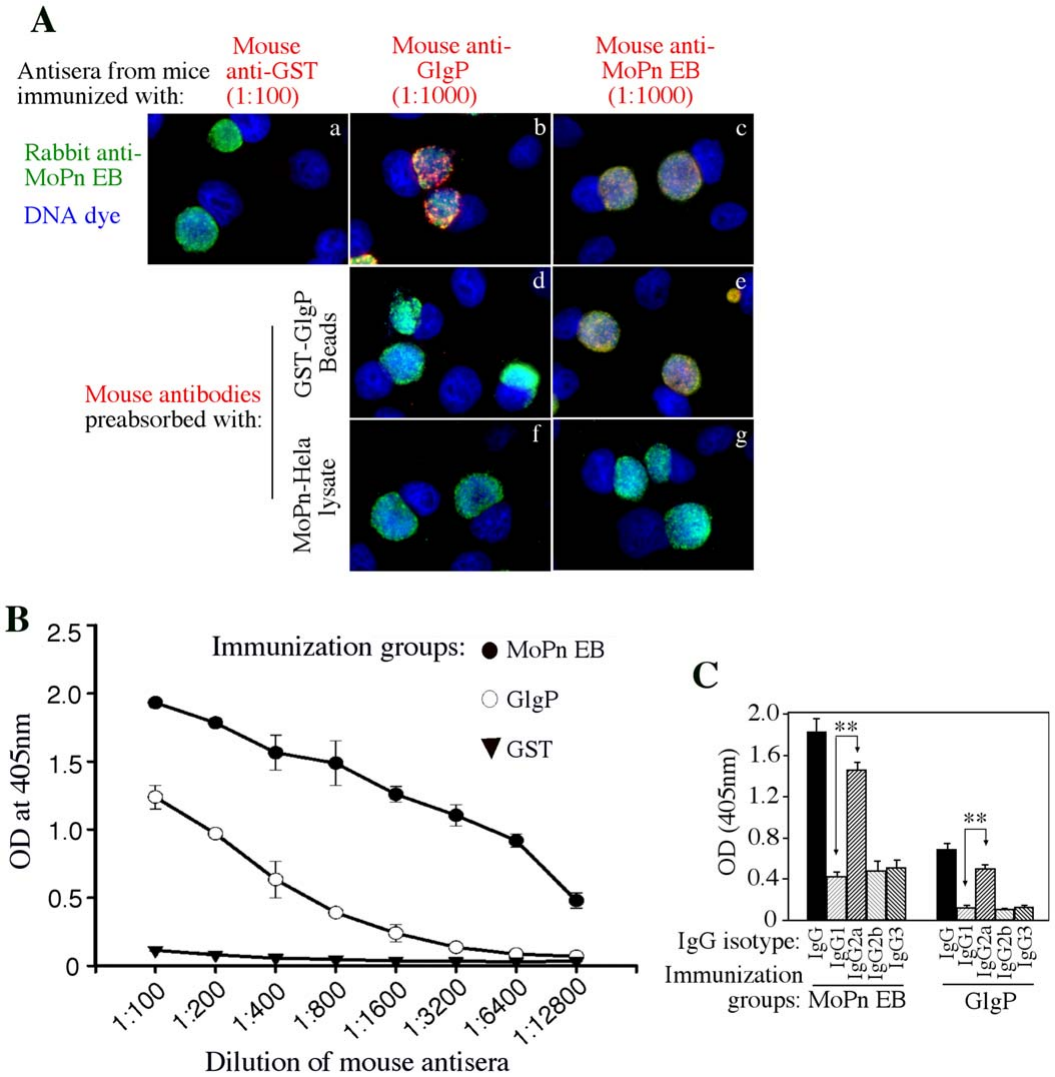
Induction of *C. muridarum*-specific immune responses with GlgP immunization. Three groups of mice with 5 in each group immunized with GST, *C. muridarum* (or MoPn) EB or GlgP as described in Fig. 1 legend were sacrificed prior to challenge infection for collecting blood and splenocytes. (**A**) The antisera after pooling from each group were used to detect the endogenous *C. muridarum* antigens in the infected cells using an immunofluorescence assay. The mouse antibody binding (red) was visualized with a goat anti-mouse Cy3 conjugate (red) and co-labeled with a rabbit anti-chlamydial organism antibody visualized with a goat anti-rabbit Cy2 conjugate (green) and a DNA dye (blue). To confirm the staining specificity, the antisera from GlgP- (panels b, d & f) and EB (panels c, e & g)-immunized groups were pre-absorbed with either GST-GlgP (panels d & e) or *C. muridarum*-infected cell lysates (f & g). The pooled anti-GlgP antisera were no longer able to label any endogenous antigens after pre-absorption with either GST-GlgP or *C. muridarum*-HeLa lysates. However, the binding to endogenous antigens by the pooled anti-EB antiserum was only blocked by *C. muridarum*-HeLa lysates. (**B**) Individual antisera from the 3 groups of mice, after serial dilution, were reacted with purified EBs coated onto ELISA plates. Antisera from the GlgP-immunized mice significantly recognized the whole organisms although with much lower titers comparing to the EB-immunized mice. (**C**) The reactivity of individual antisera from EB (1∶800 dilution) or GlgP (1∶400) -immunized mice with the whole organisms coated onto the ELISA plates was also probed with goat anti-mouse Ig isotype-specific secondary antibodies. Both EB- and GlgP-immunized mice preferentially produced higher levels of IgG2a, indicating the dominance of Th1 cytokines in these mice. ** indicates p<0.01.

**Figure 5:**
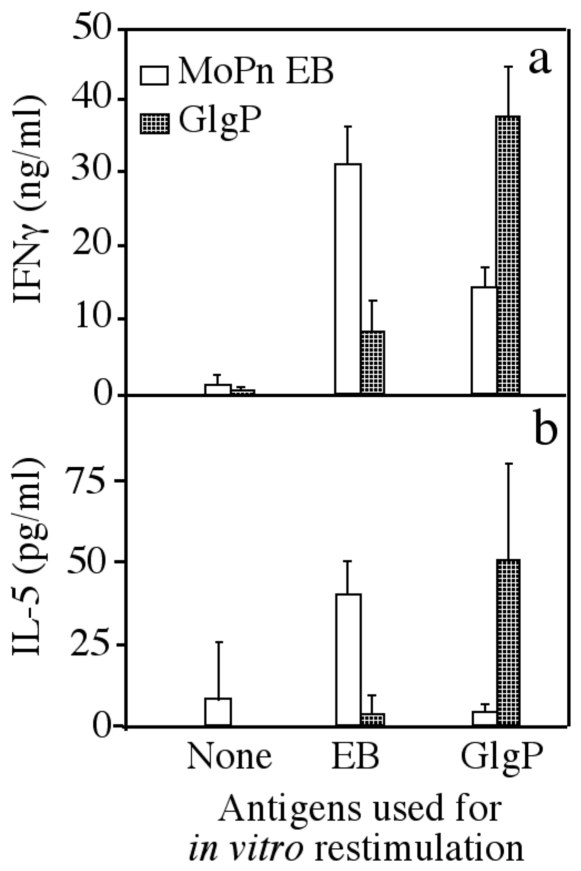
Detection of a Th1-dominant cellular response in GlgP-immunized mice. Splenocytes harvested from EB (open bar) or GlgP (hatched bar) -immunized mice as described in Fig. 4 legend were *in vitro* re-stimulated with UV-inactivated *Chlamydia muridarum* EB organisms at 1×10^6^ IFUs per well, 10 µg/ml GlgP or medium alone (none) as indicated at the bottom of the figure. Three days after the stimulation, the culture supernatants were collected for IFNg and IL-5 detection and the results were expressed as pg or ng/ml as displayed along the Y-axis (mean ± SD). Note that both EB and GlgP-immunized mice produced much higher concentrations of IFNg than IL-5, indicating a Th1 dominant cellular response in theses mice.

**Figure 6:**
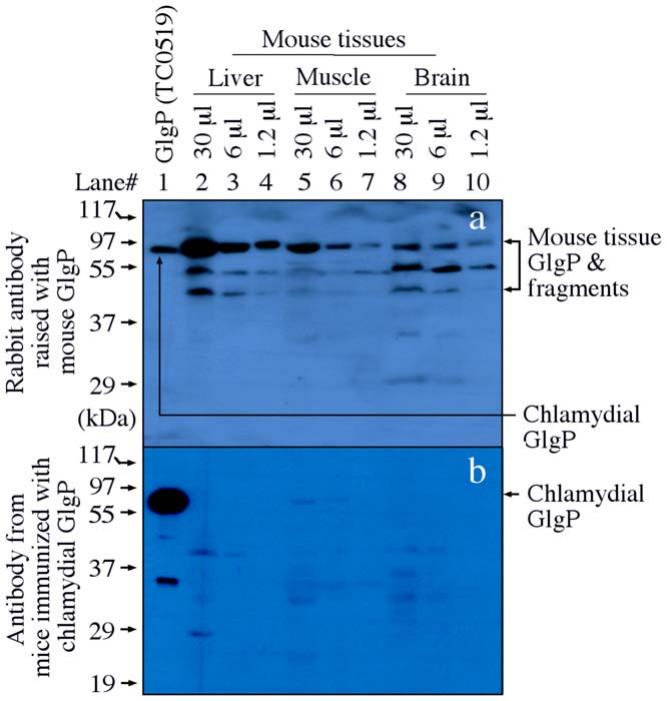
Minimal cross-reactivity of chlamydial GlgP-induced mouse antibodies with mouse tissue GlgP molecules. *C. muridarum*-infected HeLa lysate (lane 1) or extracts from mouse liver (lanes 2–4), muscle (5–7) and brain (8–10) were resolved in a SDS polyacrylamide gel and the protein bands were blotted onto nitrocellulose membrane for Western blot detection with an rabbit anti-mouse GlgP (panel a) or the pooled mouse anti-chlamydial GlgP (b). Note that the mouse anti-chlamydial GlgP antibodies preferentially recognized chlamydial GlgP without any significant cross-reactivity with mouse tissue GlgP.

The immune responses induced by GlgP immunization were monitored prior to *C. muridarum* challenge infection. Antibodies from GlgP- and EB- but not GST-immunized groups labeled *C. muridarum* inclusions in the infected HeLa cells (Fig. 4A). This immunolabeling was specific since the antibody reactivities were blocked by pre-absorption with specific antigens. The antibodies from the GlgP-immunized mice also reacted with purified *C. muridarum* EB organisms coated on the ELISA plate although the titer was not as high as that from the EB-immunized group (Fig. 4B). When the *C. muridarum*-specific antibodies were analyzed for isotypes, both EB- and GlgP-immunized mice developed high levels of IgG2a than IgG1 (Fig. 4C), indicating that both groups of mice developed a Th1 dominant T cell response. Indeed, splenocytes from both GlgP- and EB-immunized mice produced high levels of the Th1 cytokine IFNg upon *in vitro* restimulation with either EB or GlgP antigens (Fig. 5). GlgP is a highly conserved phosphorylase and the chlamydial GlgP share ∼50% amino acid identity with GlgPs from mice (NP_722476 from brain, NP_035354 from muscle or NP_573461 from liver) or human (AAC18079) (http://www.ncbi.nlm.nih.gov/blast). We then tested whether antibodies produced in mice immunized with chlamydial GlgP could cross-react with mouse GlgP (Fig. 6). Surprisingly, despite the high level of amino acid sequence homology, the anti-chlamydial GlgP antibodies did not recognize GlgP from mouse liver, brain or muscle tissues although under the same condition, the chlamydial GlgP was significantly recognized. As a control, a commercial rabbit anti-mouse GlgP antibody recognized both mouse and chlamydial GlgP.

## Discussion

To search for chlamydial vaccine candidate antigens, we used a *C. muridarum* intravaginal infection mouse model to evaluate the immunogenicity of and the ability to induce protective immunity by *C. muridarum* antigens. The *C. muridarum* antigens were selected when their counterparts in *C. trachomatis* were significantly recognized by antibodies from women urogenitally infected with *C. trachomatis* [26], [27]. Since plasmid-free chlamydial organisms failed to induce pathologies [21], [25], it is assumed that plasmid-encoded and/or regulated genes play important roles in chlamydial pathogenesis. The chlamydial plasmid encodes 8 of its own genes and regulates 22 genes encoded in the genome. Among the 22 plasmid-regulated gene products, 7 *C. trachomatis* proteins were significantly recognized by *C. trachomatis*-infected women [26]. We compared the protection efficacy of the 7 antigens using the corresponding *C. muridarum* orthologs in mice and found that chlamydial GlgP-immunized mice developed the most pronounced protective immunity against *C. muridarum* intravaginal infection with a significant reduction in vaginal shedding of live organisms on day 14 after infection. The severity of oviduct hydrosalpinx was significantly reduced in GlgP-immunized mice although the overall incidence of hydrosalpinx was not significantly different between immunized and control mice. Since the severity of hydrosalpinx can reflect the duration of tubal blockage and contribute to clinical complications such as infertility, reducing the severity of hydrosalpinx through vaccination is medically relevant.

The protective immunity induced by chlamydial GlgP is somewhat unexpected given the high level of amino acid sequence homology between chlamydal and host GlgPs. Among the 7 antigens tested, 3 are hypothetical proteins with no significant homology with any mammalian proteins. Yet, these antigens failed to induce significant protective immunity. Nevertheless, chlamydial antigens with amino acid sequence homology with their counterparts in mammalian hosts have been shown to be immunodominantly recognized by the mammalian hosts [26], [28], suggesting that the orthologous chlamydial antigens can be immunogenic in mammalian hosts. Interestingly, antibodies from the chlamydial GlgP-immunized mice failed to recognize any GlgPs in mouse tissues, demonstrating that in response to chlamydial GlgP immunization, the inbred mice selectively produced antibodies against the variable regions of GlgP and maintained tolerance to the conserved epitopes. However, it will be very difficult to predict whether similar tolerance can be maintained after the repeated injections are applied to outbred humans during human vaccination. Thus, caution needs to be taken when considering chlamydial GlgP as a vaccine antigen for humans.

The GlgP-induced partial protection correlated with GlgP-specific antibody and Th1-dominant cellular responses. Although these correlative observations cannot tell us exactly which immune responses are required for the GlgP-induced protection, evidence accumulated in the past half century has demonstrated that a Th1-dominant CD4+ T cell response is essential for controlling chlamydial urogenital infection [6], [15]. Thus, we can reasonably assume that a GlgP-specific Th1-dominant cellular response may play a critical role in GlgP-induced protective immunity, which means that GlgP has to be processed and GlgP-derived epitopes must be presented by both antigen presentation cells such as dendritic cells during immunization (for priming naïve T cells into effective/memory T cells) and the infected epithelial cells following intravaginal infection (for T cell recognition of target cells). Since GlgP is localized within the chlamydial inclusion and most likely associated with the organisms (Fig. 4A & B of current study), it would be difficult for the infected cells to access to GlgP from live organism-laden inclusions. Most likely, the newly infected cells may take up and process GlgP from dead organisms released from the burst of previously infected cells. This is possible since chlamydial inhibition of phagolysosomal fusion is only restricted to the live organism-laden inclusions/vacuoles [29]. In addition, presentation of chlamydial GlgP by nearby uninfected epithelial cells and macrophages may also provide ligands for recognition by GlgP-specific CD4+ T cells. These T cell recognitions can lead to a robust production of IFN-gamma and activation of other immune effector mechanisms at the site of infection or the site where the infection spreading occurs. IFN-g produced at high concentrations at the site of infection may significantly inhibit chlamydial replication and effectively block spreading. The dependence of GlgP-induced protection on the processing of GlgP released from already infected cells may partially explain the result that a significant reduction in live organism shedding in the GlgP-immunized group occurred only 14 days after infection but not earlier as in the live organism-immunized group. An earlier protection may be offered via T cells primed by the antigens that can be processed and presented by target cells upon an initial infection. In addition, antibody responses to organism surface-exposed epitopes may be more robust in reducing the initial infection. Since the GlgP-immunized mice produced antibodies that were able to recognize whole EBs in an ELISA, anti-GlgP antibodies may also play an important role in GlgP-induced protective immunity. However, it is not clear at this moment whether GlgP is exposed on live infectious EBs. In any case, defining the precise molecular and cellular mechanisms of GlgP-induced protective immunity needs further investigations. Regardless of the precise mechanisms involved, the current study has presented convincing evidence for a proof of concept that intramuscular immunization with purified chlamydial proteins can be used to identify vaccine candidate antigens for preventing *C. trachomatis* infection and diseases. Efforts are underway to screen other immunodominant antigens for inducing protective immunity using the *C. muridarum* intravaginal infection model. Once additional protective antigens are identified, more definitive approaches will be used for comparing their protective mechanisms. Given the complex chlamydial organism structures and chlamydial infection biology, it is likely that immunization with combinations of various antigens with each able to induce unique effector mechanisms to simultaneously attack multiple stages of the infection processes may be necessary to achieve a full protection against chlamydial infection and chlamydial diseases.

## Materials and Methods

### Ethics Statement

*The studies described in the manuscript do not involve human subjects or non-human primates, thus no institutional review board approval is required. The use of mice in the manuscript was approved by the University of Texas Health Science Center at San Antonio Animal Use Protocol Committee. The protocol number is 09053, which has been evaluated annually. The current version is valid till April 1 2012.*

### 1. Chlamydial organisms and chlamydial infection in cell culture

*C. muridarum* Nigg strain (also called MoPn) organisms were grown, purified and titrated as described previously [20]. Aliquots of the organisms were stored at −80°C till use. HeLa cells (ATCC, Manassas, VA 20108) were maintained in DMEM (GIBCO BRL, Rockville, MD) with 10% fetal calf serum (FCS; GIBCO BRL) at 37°C in an incubator supplied with 5% CO_2_. To prepare chlamydial infection samples for various assays, HeLa cells grown in tissue culture flasks or on glass coverslips in 24 well plates were pretreated with DMEM containing 30 µg/ml of DEAE-Dextran (Sigma, St Luis, MO) for 10 min. After the DEAE-Dextran solution was removed, chlamydial organisms diluted in DMEM were added to the monolayers and allowed to attach to the cell monolayers for 2 h at 37°C. The infected cells were continuously cultured in DMEM with 10% FCS and 2 µg/ml of cycloheximide (Sigma) and processed at various time points after infection as indicated in individual experiments. The infectious dose was pre-titrated and an infection rate of 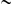
50% or less was applied to samples for immunofluorescence assays or 90% or higher for Western blot assays or making cell lysates.

### 2. Prokaryotic expression of chlamydial fusion proteins and protein purification

Seven ORFs (open reading frames) from *C. muridarum* genome were used for the current study: TC0075, TC0285, TC0419 (all three are hypothetical proteins), TC0181 (glycogen synthase or GlgA), TC0257 (glycogen branching enzyme or GlgB), TC0519 (glycogen phosphorylase, GlgP) & TC0156 (1-acyl-sn-glycerol-3-phosphate acyltransferase or AGPAT; (http://www.ncbi.nlm.nih.gov/sites/entrez). These 7 ORFs are among the 22 chromosomal genes transcriptionally regulated by the cryptic plasmid [24] and their counterparts in *C. trachomatis* were significantly recognized by *C. trachomatis*-infected women while the remaining 15 were not. The 7 ORFs were cloned into pGEX vectors (Amersham Pharmacia Biotech, Inc., Piscataway, NJ) and expressed as fusion proteins with glutathione-s-transferase (GST) fused to the N-terminus. Expression of the fusion proteins was induced with isopropyl-beta-d-thiogalactoside (IPTG; Invitrogen, Carlsbad, CA) and the fusion proteins were extracted by lysing the bacteria via sonication in a Triton-X100 lysis buffer (1% Triton-X100, 1 mM PMSF, 75 units/ml of aprotinin, 20 µM leupeptin and 1.6 µM pepstatin). After a high-speed centrifugation to remove debris, the fusion protein-containing supernatants were purified using glutathione-conjugated agarose beads (Pharmacia). While some of the bead-bound fusion proteins were used to deplete antigen-specific antibodies from antiserum samples, others were further used to purify soluble proteins by cleavage (for TC0075, TC0156 or AGPAT, TC0181 or GlgA, TC0257, TC0285 & TC0519 or GlgP) with a precision protease (Pharmacia) or by elution (TC0419) with reduced glutathione (Sigma). The cleaved or eluted chlamydial proteins were concentrated via centricon (Millipore, Billerica, MA) and used to immunize mice for antibody production or evaluating protective immunity as described previously [27], [30].

### 3. Mouse immunization and urogenital tract infection

For the initial screening experiment, female Balb/c mice were purchased at the age of 3 to 4 weeks old from Charles River Laboratories, Inc. (Wilmington, MA) and divided into 9 different groups with 5 in each group. All mice were immunized intramuscularly (i.m.) for a total of 3 times on day 0, day 20 and day 30 respectively. A total of 30 µg protein antigen or 10^6^ IFUs of *C. muridarum* (or MoPn) EBs plus 10 µg of CpG in 50 µl PBS emulsified in 50 µl of IFA (incomplete Freund's Adjuvant, Sigma-Aldrich, St. Luis, MO) was given to each mouse at each time. The first group was given GST protein as negative and the second group with EBs as positive control. The remaining 7 groups were each immunized with one of the 7 test antigens. The CpG with a sequence of 5′-TCC.ATG.ACG.TTC.CTG.ACG.TT-3′ (all nucleotides are phosphorothioate-modified at the 3′ internucleotide linkage) was used (Integrated DNA Technologies, IDT, Coralville, IA). We used the CpG-IFA as adjuvant for the intramuscular injection because it has been previously shown to induce Th1 dominant immune responses. Thirty days after the third immunization, each mouse was inoculated intravaginally with 2×10^4^ IFUs of live *C. muridarum* organisms in 20 µl of SPG. Five days prior to infection, each mouse was injected with 2.5 mg Depo-provera (Pharmacia Upjohn, Kalamazoo, MI) subcutaneously. One day before the infection, all mice were bled from the tail for monitoring antibody responses. In some experiments, mice were sacrificed prior to infection for monitoring both antibody and cellular responses. The GlgP immunization experiment was repeated several times with a total of 15 to 17 mice per group.

### 4. Monitoring mouse vaginal live chlamydial organism shedding and evaluating upper genital tract pathologies

To monitor live organism shedding, vaginal swabs were collected weekly for 4 weeks after intravaginal infection. Each swab was soaked in 0.5 ml of SPG, and after vortexing, the chlamydial organisms released into the supernatants were titrated on HeLa cell monolayers in duplicates as described previously [19], [20], [27]. Briefly, serially diluted swab samples were inoculated onto HeLa cell monolayers grown on coverslips in 24 well plates. After incubation for 24 hours in the presence of 2 µg/ml cycloheximide, the cultures were processed for immunofluorescence assay as described below. The inclusions were counted under a fluorescence microscope. Five random fields were counted per coverslip. The total number of IFUs per swab was calculated based on the number of IFUs per field, number of fields per coverslip, dilution factors and inoculation and total sample volumes. An average was taken from the serially diluted and duplicate samples for any given swab. The calculated total number of IFUs/swab was converted into log_10_ and the log_10_ IFUs were used to calculate means and standard deviation for each group at each time point. Mice were sacrificed 60 days after infection and the mouse urogenital tract tissues were isolated for gross pathology evaluation as described previously [19], [20]. Both incidence and severity of hydrosalpinx were recorded for each mouse and compared between different groups. The severity of hydrosalpinx was scored based on the following criteria: No hydrosalpinx is assigned a score of zero (0); Hydrosalpinx is only visible after amplification (1); Hydrosalpinx is clearly visible with naked eye but the size is smaller than that of ovary (2); The size of hydrosalpinx is similar to that of ovary (3); If larger than ovary (4). Apparently, the hydrosalpinx severity (scored based on the size of the affected oviduct) largely reflects the amounts of fluids accumulated in the oviduct. The fluid accumulation may be impacted by both the duration of the oviduct blockage and the extent of the inflammatory responses in the oviduct. Regardless how the fluids are accumulated, the severity of hydrosalpinx has clinical significance. Numerous clinical studies have shown that women with more severe tubal damage or larger hydrosalpinges have statistically significant lower rates of pregnancy and live birth following in vitro fertilization (IVF) and surgical treatment of severe hydrosalpinages prior to IVF can significantly increase the live birth rate [31], [32], [33], [34]. Thus, scoring the hydrosalpinx severity is a medically relevant measurement.

### 5. Immunofluorescence assay

HeLa cells grown on glass coverslips in 24 well plates with or without chlamydial organisms were fixed with 2% paraformaldehyde for 30 min, followed by permeabilization with 2% saponin (Sigma) for an additional 1 h. After washing and blocking, the cell samples were labeled with Hoechst (blue, Sigma) for visualizing DNA and a rabbit anti-chlamydial chaperon cofactor antibody (unpublished data) plus a goat anti-rabbit IgG conjugated with Cy2 (green; Jackson ImmunoResearch Laboratories, Inc., West Grove, PA) for visualizing chlamydial inclusions. In some experiments, the monolayers were co-stained with mouse anti-chlamydial protein antibodies plus a goat anti-mouse IgG conjugated with Cy3 (red; Jackson ImmunoResearch Laboratories). The immuno-labeled cell samples were quantitated as described above and used for image analysis and acquisition with an Olympus AX-70 fluorescence microscope equipped with multiple filter sets (Olympus, Melville, NY) as described previously [35], [36], [37], [38]. All microscopic images were processed using the Adobe Photoshop program (Adobe Systems, San Jose, CA).

### 6. Enzyme-linked immunosorbent assay (ELISA)

The cytokines in the supernatants of the *in vitro* stimulated lymphocyte cultures were measured using standard cytokine ELISA kits (mouse IFNg kit, cat# DY485 & IL-5 (cat# DY405, both from R&D Systems, Inc., Minneapolis, MN) as instructed by the manufacturer and described previously [19], [39]. Briefly, splenocytes were harvested from immunized mice prior to MoPn infection and stimulated *in vitro* with UV-inactivated MoPn EBs, chlamydial GlgP or medium alone for 3 days. The culture supernatants were collected for cytokine measurements using 96 well ELISA microplates precoated with the corresponding capture antibodies. The capture antibody-bound cytokines were detected with biotin-conjugated antibodies and horseradish peroxidase (HRP)-conjugated Avidin. The cytokine concentrations were calculated based on absorbance values, cytokine standards and sample dilution factors and expressed as ng or pg per ml.

### 7. Western blot assay

The Western blot assay was carried out as described elsewhere [40], [41]. Briefly, either the *C. muridarum* GlgP or mouse tissue samples were solubilized in 2% SDS sample buffer and loaded to a SDS–polyacrylamide gel. After electrophoresis, the resolved protein bands were transferred to nitrocellulose membranes for blotting with primary antibodies, including the mouse pAb against *C.muridarum* GlgP or a rabbit anti-mouse liver GlgP antibody (Rabbit pAb against liver glycogen phosphorylase, cat#15851-1-AP, ProteinTech Group, Chicago, IL) and the mouse pAb against *C.muridarum* TC0519. The primary antibody bindings were probed with an HRP (horse radish peroxidase)-conjugated goat anti-mouse or rabbit secondary antibodies and visualized with an enhanced chemiluminescence (ECL) kit (Santa Cruz Biotechnology, Inc., Santa Cruz, CA). The various tissue samples harvested from mouse brain, liver or muscle were homogenized in ice-cold buffer containing 50 mM HEPES (pH 7.6), 150 mM sodium chloride, 20 mM sodium pyrophosphate, 20 mM beta-glycerophosphate, 10 mM sodium fluoride, 2 mM sodium orthovanadate, 2 mM EDTA, 1.0% Igepal (a nonionic, nondenaturing detergent), 10% glycerol, 2 mM phenylmethylsulfonyl fluoride, 1 mM magnesium chloride, 1 mM calcium chloride, 10 µg/ml leupeptin, and 10 ìg/ml aprotinin. Tissue homogenates were centrifuged and the supernatants were resolved in SDS-polyacrylamide gel and blotted onto nitrocellulose membrane for antibody detection.

### 8. Statistical analysis

ANOVA test (http://www.physics.csbsju.edu/stats/anova.html) was performed to analyze data from multiple groups and a two-tailed Student's *t*-test (Microsoft Excel) to compare the means between two groups. A Fisher's Exact test was used for comparing the incidences between two groups.
